# Burden of Bowel Urgency in Patients With Ulcerative Colitis and Crohn’s Disease: A Real-World Global Study

**DOI:** 10.1093/crocol/otae047

**Published:** 2024-08-10

**Authors:** Raja Atreya, Isabel Redondo, Petra Streit, Marijana Protic, Susanne Hartz, Gamze Gurses, Hannah Knight, Sophie Barlow, Niamh Harvey, Theresa Hunter Gibble

**Affiliations:** Medical Department 1, University Hospital Erlangen, Friedrich Alexander University Erlangen-Nürnberg, Erlangen, Germany; Medical Affairs, Eli Lilly and Company, Lisbon, Portugal; Global New Product Planning, Eli Lilly and Company, Vernier, Switzerland; Medical Affairs, Eli Lilly and Company, Vernier, Switzerland; Value, Evidence and Outcomes – International, Eli Lilly and Company, Bracknell, UK; Reimbursement Access, Eli Lilly and Company, Istanbul, Turkey; Autoimmune Franchise, Adelphi Real World, Bollington, UK; Statistics & Data Analytics, Adelphi Real World, Bollington, UK; Autoimmune Franchise, Adelphi Real World, Bollington, UK; Value, Evidence and Outcomes, Eli Lilly and Company, Indianapolis, USA

**Keywords:** bowel urgency, Crohn’s disease, quality of life, ulcerative colitis

## Abstract

**Background:**

Bowel urgency is a highly disruptive and bothersome symptom experienced by patients with inflammatory bowel diseases (IBD), (ulcerative colitis [UC], and Crohn’s disease [CD]). However, the burden of bowel urgency among patients with varying experiences in targeted treatment has not been consistently assessed. This real-world study explored the clinical and health-related quality of life burden of bowel urgency among patients with IBD with differing treatment experiences.

**Methods:**

This cross-sectional survey included gastroenterologists and their patients with IBD across France, Germany, Italy, Spain, the United Kingdom, and the United States treated for over 3 months. Physicians provided patient demographics, clinical characteristics, and treatment history. Patients reported their health-related quality of life and work productivity. Patients with UC and CD were analyzed separately and stratified into 3 groups: Targeted therapy naïve, those receiving their first-line targeted therapy, and targeted therapy experienced.

**Results:**

This study found that 17%-26% of UC and 13%-17% of CD patients experienced persistent bowel urgency, irrespective of receiving conventional or targeted therapy. Moreover, patients with bowel urgency experienced an increased clinical and health-related quality of life burden compared to patients without bowel urgency, which physicians most commonly regarded as one of the most difficult symptoms to treat, with the burden remaining substantial irrespective of their treatment experience.

**Conclusions:**

Despite several current treatment options, new therapeutic strategies are necessary to provide relief from bowel urgency, one of the most challenging symptoms for people living with IBD.

## Graphical Abstract

**Graphical Abstract:**
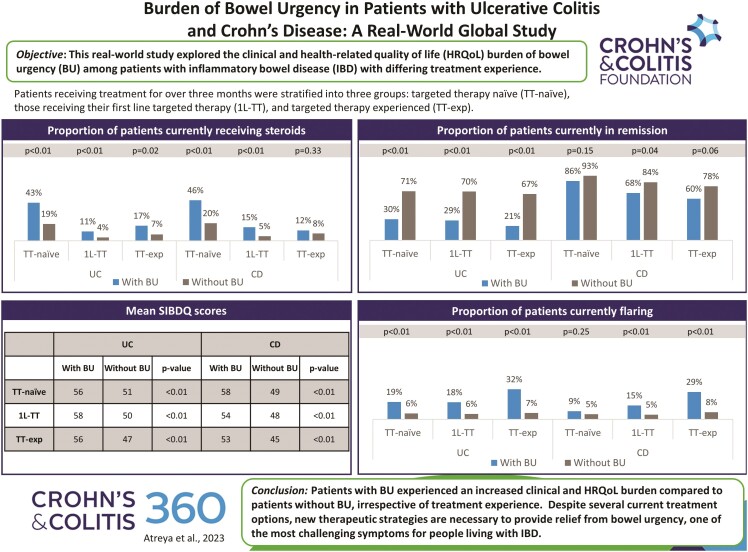

## Introduction

Inflammatory bowel diseases (IBD) include ulcerative colitis (UC) and Crohn’s disease (CD), which are chronic, idiopathic, immune-mediated disorders characterized by inflammation and tissue damage of the gastrointestinal tract.^1–3^ Common symptoms of IBD include chronic diarrhea, abdominal pain, gastrointestinal bleeding, weight loss, and malnutrition.^4^ The symptoms of abnormal anorectal function, such as bowel urgency, tenesmus, and fecal incontinence (also referred to as urge or bowel incontinence, defined as the inability to control bowel movements),^5,6^ are also prevalent in patients with IBD, regardless of the presence or absence of perianal disease.

Bowel urgency, also referred to as urgency, rectal urgency, or fecal urgency, is the sudden need for a bowel movement,^7^ and is one of the most important, disruptive, and bothersome symptoms experienced by patients with IBD, especially when associated with fecal incontinence.^8–12^ Bowel urgency and fear of fecal incontinence have been reported to be the main symptoms leading to patients with UC declining participation in work/school, social events, and sports/physical exercise. Previous studies have also shown bowel urgency to be associated with higher disease activity, decreased work productivity, and markedly reduced health-related quality of life (HRQoL) in patients with IBD.^13,14^

While professional clinical guidelines recognize bowel urgency as an important disease-related symptom for patients with active UC,^15–17^ it has only very recently been agreed that bowel urgency should be captured as a core outcome in UC, given patient input on the debilitating nature of this symptom.^18^ Prior to this recent change in recommendations, the assessment of bowel urgency has not been a recommended measured endpoint in clinical trials for either UC or CD and, therefore, is not consistently assessed.^19^ Moreover, the usual disease assessment measures of UC and CD often omit key symptoms, including bowel urgency and fecal incontinence, that significantly impact patients’ HRQoL and everyday lives,^9,10,20^ so important symptoms may be missed during clinical assessments.

Current treatment options for IBD include conventional therapies (5-aminosalicylates [5-ASAs], steroids, immunomodulators) and targeted therapies (TT) such as anti-tumor necrosis factor monoclonal antibodies (anti-TNFs), interleukin 12/23 inhibitors, integrin receptor antagonists, Janus kinase (JAK) inhibitors, and sphingosine-1-phosphate (S1P) receptor modulators.^21,22^ The advent of TT has substantially improved therapeutic outcomes and made a major impact on existing therapeutic algorithms.^23^ However, one of the main problems associated with existing therapies, in particular biologic therapies, is the considerable rate of secondary loss of response which prevents many patients from achieving stable, durable remission and, therefore, exposes them to tissue damage progression associated with disease activity.^24,25^

### Aim of the Study

Given that the burden of bowel urgency among patients has not been consistently assessed, coupled with the number of new therapeutic options now currently available, the objective of this study was to assess the clinical and HRQoL burden of bowel urgency among patients with UC and CD with differing levels of TT experience.

## Materials and Methods

### Study Design

Data were extracted from the Adelphi Inflammatory Bowel Disease (IBD) Disease Specific Program (DSP)™, a cross-sectional survey of gastroenterologists and their consulting patients presenting in a real-world clinical setting, conducted between January 2020–March 2021 in France, Germany, Italy, Spain, the United Kingdom, and the United States. DSPs are large, multinational, observational studies collecting information on real-world clinical practice, designed to identify current disease management and patient- and physician-reported disease impact.^26^ The DSP methodology has been previously published and validated.^26–28^

A geographically representative sample of physicians (*n* = 346) were recruited to participate in the DSP by local fieldwork agencies following the completion of a short screening questionnaire, with physicians eligible to participate provided they were personally responsible for treatment decisions and management for a minimum of 5 patients with UC and 5 patients with CD in a typical month. The data collection setting was secondary gastroenterology services (public or private hospitals, clinics, or offices). Physician participation was financially incentivized, with reimbursement upon survey completion according to fair market research rates.

Physicians were instructed to complete a patient record form for their next 5-7 consecutively consulting patients with UC or 5-8 with CD under routine care. Patients were eligible for inclusion if aged ≥18 years, with a physician-confirmed diagnosis of UC or CD and were not involved in clinical trials. Given this study assessed the clinical and HRQoL burden of bowel urgency among patients with differing levels of TT experience, UC patients were excluded from the study if they had only ever had a history of mild disease (had never received a steroid, immunomodulator or biologic, and had never had a Mayo score >4). We applied no such further inclusion/exclusion criteria in patients with CD due to the progressive nature of the disease. Furthermore, existing data pertaining to the burden of bowel urgency and its presentation by TT experience in patients with CD is lacking, and hence there is a need to generate such real-world evidence for patients with CD, regardless of disease presentation.

This physician-reported patient record form contained detailed questions on patients’ demographics, clinical assessments, clinical outcomes, and treatment history. To assess bowel urgency, physicians were asked to select the symptoms relevant to the question “Which of the following symptoms is the patient currently experiencing?”. Bowel urgency was evaluated by physicians checking the boxes for “Bowel movement urgency” and/or “Night-time bowel movement urgency.” Physicians reported their assessment of patients’ disease severity (mild, moderate, or severe) based on their own clinical judgment. Remission status was based on a Mayo score <3 for patients with UC, calculated through collection of the Mayo score stool frequency, rectal bleeding, Physician Global Assessment, and endoscopic subscore components (based on the most recent endoscopic observation),^29^ and a Crohn’s Disease Activity Index (CDAI) score <150 for patients with CD.^30^ Completion of the physician-reported patient record form was undertaken through consultation of existing patient clinical records, as well as the judgment and diagnostic skills of the respondent physician, which is consistent with decisions made in routine clinical practice.

Each patient for whom the physician completed a patient record form was then invited to voluntarily complete a self-reported questionnaire and, upon agreement, provided their informed consent to participate. The questionnaire included validated instruments relating to the impact of the patient’s condition on their HRQoL, including the EuroQol Visual Analogue Scale (EQ VAS),^31^ the Short Inflammatory Bowel Disease Questionnaire (SIBDQ),^32^ and the Work Productivity and Activity Impairment (WPAI) questionnaire.^33^

Patient-reported questionnaire forms were completed by the patient independently from their physician and returned in a sealed envelope ensuring their responses were kept confidential. Patients were not compensated for participation.

### Data Analysis

Patients were stratified into those who were currently receiving conventional therapy with a duration >3 months and had never received TT (TT-naïve), patients who were currently receiving their first TT with a duration >3 months (1L-TT), and patients currently receiving their second or later TT with duration >3 months (TT-exp), in order to understand whether the burden of bowel urgency differs based on treatment experience. Patients were further stratified by the presence or absence of bowel urgency (day or night-time) at the time of data collection, in order to identify the burden of bowel urgency among patients within specific treatment groups. Data from patients with UC and CD were presented separately, given these are 2 distinct entities of IBD with differing symptomatic burdens and treatment considerations.

As the primary objective of the survey was descriptive (ie, no a priori hypotheses specified), the sample size was fixed by the duration of the survey period. Therefore, formal sample size calculations were not applicable and were not performed. Continuous data were expressed as means and SD and compared using a *t*-test, while categorical variables, summarized by frequencies and proportions, were compared using Fisher’s exact, Mann–Whitney, or χ^2^ tests, as appropriate. Missing data were not imputed, such that the base of patients for analysis could vary from variable to variable. Analyses were conducted in Stata Statistical Software 17.^34^

### Ethical Considerations

Using a checkbox, patients provided informed consent to take part in the survey. Data were collected in such a way that patients and physicians could not be identified directly. Physician and patient data were pseudo-anonymized. A code was assigned when data were collected. Upon receipt by Adelphi Real World, data were pseudo-anonymized again to mitigate against tracing them back to the individual. Data were aggregated before being shared with the subscriber and/or for publication.

This research was submitted to the Western Institutional Review Board, study protocol number 1-1238963-1. Data collection were undertaken in line with European Pharmaceutical Marketing Research Association guidelines^35^ and as such it did not require ethics committee approval. Each survey was performed in full accordance with relevant legislation at the time of data collection, including the US Health Insurance Portability and Accountability Act 1996,^36^ and Health Information Technology for Economic and Clinical Health Act legislation.^37^

## Results

### Ulcerative Colitis

Data were provided by 346 gastroenterologists for 2259 eligible patients with UC (France; *n* = 381 [17%], Germany; *n* = 404 [18%], Italy; *n* = 376 [17%], Spain; *n* = 408 [18%], United Kingdom; *n* = 207 [9%] and the United States; *n* = 483 [21%]). Patients were stratified into 3 treatment groups and further categorized into patients with reported bowel urgency and those with no bowel urgency. Despite receiving treatment for more than 3 months, physicians reported that 23%, 17%, and 26% of TT-naïve, 1L-TT, and TT-exp patients, respectively, were experiencing bowel urgency as a current symptom (Table 1).

**Table 1.** Demographic and disease characteristics of patients with ulcerative colitis by bowel urgency status and treatment experience.

	TT-naïve			1L-TT			TT-exp		
Variable	No BU (*n* = 457)	BU (*n* = 138)	*P*-value	No BU (*n* = 708)	BU (*n* = 143)	*P*-value	No BU (*n* = 238)	BU (*n* = 83)	*P*-value
*Physician-reported*									
Age, mean (SD)	40.9 (14.6)	37.8 (13.9)	.028 (*t*-test)	40.3 (13.9)	43.0 (14.5)	.037 (*t*-test)	43.5 (14.7)	42.7 (13.4)	.649 (*t*-test)
Sex, male, *n* (%)	264 (57.8)	67 (48.6)	.063 (FE)	396 (55.9)	71 (49.7)	.197 (FE)	118 (49.6)	46 (55.4)	.375 (FE)
BMI, mean (SD)	24.2 (3.4)	23.2 (3.0)	.003 (*t*-test)	24.7 (3.7)	24.9 (4.4)	.499 (*t*-test)	24.3 (3.9)	24.6 (4.5)	.557 (*t*-test)
*Smoking status, n*	424	133	.001 (CH)	667	136	.067 (CH)	228	75	.515 (CH)
Current smoker, *n* (%)	72 (17.0)	35 (26.3)		79 (11.8)	19 (14.0)		24 (10.5)	5 (6.7)	
Ex-smoker, *n* (%)	108 (25.5)	45 (33.8)		193 (28.9)	51 (37.5)		78 (34.2)	24 (32.0)	
Never smoked, *n* (%)	244 (57.5)	53 (39.8)		395 (59.2)	66 (48.5)		126 (55.3)	46 (61.3)	
*Employment status, n*	436	137	<.001 (CH)	684	140	.601 (CH)	231	78	.896 (CH)
Working full-time, *n* (%)	284 (65.1)	68 (49.6)		400 (58.5)	72 (51.4)		125 (54.1)	43 (55.1)	
Working part-time, *n* (%)	32 (7.3)	26 (19.0)		84 (12.3)	18 (12.9)		29 (12.6)	12 (15.4)	
On long-term sick leave, *n* (%)	6 (1.4)	1 (0.7)		11 (1.6)	3 (2.1)		12 (5.2)	4 (5.1)	
Homemaker, *n* (%)	22 (5.0)	9 (6.6)		44 (6.4)	13 (9.3)		20 (8.7)	7 (9.0)	
Student, *n* (%)	41 (9.4)	21 (15.3)		64 (9.4)	13 (9.3)		14 (6.1)	3 (3.8)	
Retired, *n* (%)	37 (8.5)	7 (5.1)		53 (7.7)	16 (11.4)		26 (11.3)	6 (7.7)	
Unemployed, *n* (%)	14 (3.2)	5 (3.6)		28 (4.1)	5 (3.6)		5 (2.2)	3 (3.9)	
Duration of IBD, *n*	399	122	.505 (*t*-test)	661	131	.204 (*t*-test)	220	72	.731 (*t*-test)
Duration of IBD, years, mean (SD)	4.8 (6.5)	4.4 (6.0)		4.8 (5.5)	5.5 (6.0)		7.8 (5.9)	7.5 (5.4)	
Current severity, *n*	457	138	<.001 (MW)	708	143	<.001 (MW)	238	83	<.001 (MW)
Mild, *n* (%)	377 (82.5)	78 (56.5)		565 (79.8)	72 (50.3)		179 (75.2)	39 (47.0)	
Moderate/severe, *n* (%)	80 (17.5)	60 (43.5)		143 (20.2)	71 (49.7)		59 (24.8)	44 (53.0)	
Current remission status, *n*	457	138	<.001 (MW)	708	143	<.001 (MW)	238	83	<.001 (MW)
In remission, *n* (%)	324 (70.9)	42 (30.4)		498 (70.3)	41 (28.7)		159 (66.8)	17 (20.5)	
Current flaring status, *n*	411	129	<.001 (FE)	670	137	<.001 (FE)	224	81	<.001 (FE)
Flaring, *n* (%)	26 (6.3)	24 (18.6)		39 (5.8)	25 (18.2)		15 (6.7)	26 (32.1)	
Current steroid use, n	457	138	<.001 (FE)	708	143	.002 (FE)	238	83	.016 (FE)
Receiving steroids, *n* (%)	87 (19.0)	59 (42.8)		27 (3.8)	15 (10.5)		17 (7.1)	14 (16.9)	
HCP visits, *n*	456	138	.001 (*t*-test)	699	143	<.001 (*t*-test)	238	83	.185 (*t*-test)
HCP visits in past year, mean (SD)	3.9 (3.2)	5.0 (4.2)		5.7 (4.5)	7.2 (6.8)		7.4 (6.6)	6.4 (4.1)	
*Patient-reported*									
EQ VAS, *n*	151	53	.007 (*t*-test)	209	52	<.001 (*t*-test)	65	24	.001 (*t*-test)
EQ VAS, mean (SD)	80.8 (11.8)	75.2 (15.5)		84.1 (14.8)	75.8 (16.8)		80.0 (15.4)	67.3 (17.6)	
WPAI, *n*	75	32	<.001 (*t*-test)	108	28	.039 (*t*-test)	38	15	.004 (*t*-test)
Overall work impairment, mean (SD) %	15.0 (19.5)	33.7 (26.5)		13.7 (20.9)	22.9 (20.5)		14.1 (16.0)	32.1 (26.7)	
SIBDQ, *n*	149	51	.004 (*t*-test)	201	52	<.001 (*t*-test)	64	24	.003 (*t*-test)
SIBDQ total score, mean (SD)	56.1 (11.5)	50.8 (10.1)		57.7 (11.0)	49.7 (10.7)		56.0 (12.4)	46.8 (14.0)	

Abbreviations: BMI, body mass index; BU, bowel urgency; CH, χ^2^ test; EQ VAS, EuroQol visual analog scale; FE, Fisher’s exact test; HCP, healthcare practitioner; IBD, inflammatory bowel disease; MW, Mann–Whitney test; SD, standard deviation; SIBDQ, Short Inflammatory Bowel Disease Questionnaire; TT-naïve, patients who were currently receiving conventional therapy with a duration >3 months and had never received targeted therapy; 1L-TT, patients who were currently receiving their first targeted therapy with a duration >3 months; TT-exp, patients who were currently receiving their second or later targeted therapy with duration >3 months; WPAI, work productivity and activity impairment.

### Comparison of Patients With and Without Bowel Urgency

Minimal differences were observed in patient demographics and disease characteristics when comparing patients with and without bowel urgency across the treatment groups (Table 1). Among TT-naïve patients, those with bowel urgency were younger (mean [SD] 37.8 [13.9] vs 40.9 [14.6], *P* = .03) and had lower mean body mass index (BMI) compared to patients without bowel urgency (23.2 [3.0] vs 24.2 [3.4], *P* < .01). A significant difference in employment status was observed within the TT-naïve group, with 50% and 65% of patients with and without bowel urgency working full-time, respectively (*P* < .01). Very few differences were observed in the 1L-TT and TT-exp groups, with no significant difference in disease duration for patients with and without bowel urgency across all 3 treatment groups.

Across the treatment groups, higher rates of moderate to severe disease activity were present among patients with versus without bowel urgency (TT-naïve; 44% vs 18%, 1L-TT; 50% vs 20%, and TT-exp; 53% vs 25%, respectively, *P* < .01; Table 1). Patients with bowel urgency across all treatment groups were consistently significantly less likely to be in remission (TT-naïve; 30%, vs 71%, 1L-TT;29% vs 70%, TT-exp; 20% vs 67%, all *P* < .01), more likely to be currently flaring (TT-naïve; 19% vs 6%, 1L-TT; 18% vs 6%, TT-exp; 32% vs 7%, all *P* < .01), and more likely to be receiving steroids (TT-naïve; 43% vs 19%, *P* < .01, 1L-TT; 10% vs 4%, *P* < .01, TT-exp; 17% vs 7%, *P* = .02), than those without bowel urgency (Table 1). Among the TT-naïve and 1L-TT groups, patients with bowel urgency had a significantly higher mean number of healthcare practitioner (HCP) visits in the past year compared to patients without bowel urgency (TT-naïve; 5.0 [4.2] vs 3.9 [3.2], 1L-TT; 7.2 [6.8] vs 5.7 [4.5], both *P* < .01; Table 1). Irrespective of treatment experience, patients with bowel urgency had significantly lower HRQoL than patients without bowel urgency as adjudged by mean scores on the EQ-VAS (TT-naïve; 75.2 [15.5] vs 80.8 [11.8], 1L-TT; 75.8 [16.8] vs 84.1 [14.8], TT-exp; 67.3 [17.6] vs 80.0 [15.4], all *P* < .01) and SIBDQ (TT-naïve; 50.8 [10.1] vs 56.1 [11.5], 1L-TT; 49.7 [10.7] vs 57.7 [11.0], TT-exp; 46.8 [14.0] vs 56.0 [12.4], all *P* < .01), and reported higher mean levels of overall work impairment (TT-naïve; 33.7 [26.5] vs 15.0 [19.5], *P* < .01, 1L-TT; 22.9 [20.5] vs 13.7 [20.9], *P* = .04, TT-exp; 32.1 [26.7] vs 14.1 [16.0], *P* < .01; Table 1).

### Comparison of Treatment Groups in Patients With Bowel Urgency

Among patients with bowel urgency, there were significant differences across the different treatment groups in patients’ mean age (37.8 [13.9] TT-naïve, 43.0 [14.5] 1L-TT, 42.7 [13.4] TT-exp, *P* < .01) and disease duration (4.4 [6.0] TT-naïve, 5.5 [6.0] 1L-TT, 7.5 [5.4] TT-exp, years, *P* < .01; Table 2). No significant differences were observed between the groups for patient sex or employment status.

**Table 2.** Physician-reported patient characteristics and patient-reported outcomes in patients with ulcerative colitis and bowel urgency by treatment experience.

Variable	TT-naive (*n* = 138)	1L-TT (*n* = 143)	TT-exp (*n* = 83)	*P*-value
*Physician-reported*				
Age, mean (SD)	37.8 (13.9)	43.0 (14.5)	42.7 (13.4)	.004 (*t*-test)
Sex, male, *n* (%)	67 (48.6)	71 (49.7)	46 (55.4)	.590 (FE)
BMI, mean (SD)	23.2 (3.0)	24.9 (4.4)	24.6 (4.5)	.001 (*t*-test)
*Smoking status, n*	133	136	75	.002 (CH)
Current smoker, *n* (%)	35 (26.3)	19 (14.0)	5 (6.7)	
Ex-smoker, *n* (%)	45 (33.8)	51 (37.5)	24 (32.0)	
Never smoked, *n* (%)	53 (39.8)	66 (48.5)	46 (61.3)	
*Employment status, n*	137	140	78	.152 (CH)
Working full-time, *n* (%)	68 (49.6)	72 (51.4)	43 (55.1)	
Working part-time, *n* (%)	26 (19.0)	18 (12.9)	12 (15.4)	
On long-term sick leave, *n* (%)	1 (0.7)	3 (2.1)	4 (5.1)	
Homemaker, *n* (%)	9 (6.6)	13 (9.3)	7 (9.0)	
Student, *n* (%)	21 (15.3)	13 (9.3)	3 (3.9)	
Retired, *n* (%)	7 (5.1)	16 (11.4)	6 (7.7)	
Unemployed, *n* (%)	5 (3.6)	5 (3.6)	3 (3.8)	
*Duration of IBD, n*	122	131	72	.002 (*t*-test)
Duration of IBD, years, mean (SD)	4.4 (6.0)	5.5 (6.0)	7.5 (5.4)	
*Current severity, n*	138	143	83	.346 (MW)
Mild, *n* (%)	78 (56.5)	72 (50.3)	39 (47.0)	
Moderate/severe, *n* (%)	60 (43.5)	71 (49.7)	44 (53.0)	
*Current remission status, n*	138	143	83	.253 (MW)
In remission, *n* (%)	42 (30.4)	41 (28.7)	17 (20.5)	
*Current flaring status, n*	129	137	81	.032 (FE)
Flaring, *n* (%)	24 (18.6)	25 (18.2)	26 (32.1)	
*Current steroid use, n*	138	143	83	<.001 (FE)
Receiving steroids, *n* (%)	59 (42.8)	15 (10.5)	14 (16.9)	
*HCP visits, n*	138	143	83	.002 (*t*-test)
HCP visits in past year, mean (SD)	5.0 (4.2)	7.2 (6.8)	6.4 (4.1)	
*Patient-reported*				
EQ VAS, *n*	53	52	24	.090 (*t*-test)
EQ VAS, mean (SD)	75.2 (15.5)	75.8 (16.8)	67.3 (17.6)	
WPAI, *n*	32	28	15	.214 (*t*-test)
Overall work impairment, mean (SD) %	33.7 (26.5)	22.9 (20.5)	32.1 (26.7)	
SIBDQ, *n*	51	52	24	.341 (*t*-test)
SIBDQ total score, mean (SD)	50.8 (10.1)	49.7 (10.7)	46.8 (14.0)	

Abbreviations: BMI, body mass index; BU, bowel urgency; CH, χ^2^ test; EQ VAS, EuroQol visual analog scale; FE, Fisher’s exact test; HCP, healthcare practitioner; IBD, inflammatory bowel disease; MW, Mann–Whitney test; SD, standard deviation; SIBDQ, Short Inflammatory Bowel Disease Questionnaire; TT-naïve, patients who were currently receiving conventional therapy with a duration >3 months and had never received targeted therapy; 1L-TT, patients who were currently receiving their first targeted therapy with a duration >3 months; TT-exp, patients who were currently receiving their second or later targeted therapy with duration >3 months; WPAI, work productivity and activity impairment.

Regardless of treatment experience, the proportion of patients with moderate to severe disease remained high among patients with bowel urgency (43% TT-naïve, 50% 1L-TT, 53% TT-exp, *P* = .35), with only 30%, 29%, and 20% of TT-naïve, 1L-TT and TT-exp patients in remission, respectively (*P* = .25; Table 2). A high proportion of patients with bowel urgency were currently flaring (19% TT-naïve, 18% 1L-TT, 32% TT-exp, *P* = .03), and 43% of TT-naïve, 10% of 1L-TT and 17% of TT-exp patients were receiving steroids (*P* < .01). The mean number of HCP visits in the past year was consistently high across patients with bowel urgency (5.0 [4.2] TT-naïve, 7.2 [6.8] 1L-TT, 6.4 [4.1] TT-exp, *P* < .01). Similarly high HRQoL, as measured by mean EQ VAS (75.2 [15.5] TT-naïve, 75.8 [16.8] 1L-TT, 67.3 [17.6] TT-exp, *P* = .09) and SIBDQ (50.8 [10.1] TT-naïve, 49.7 [10.7] 1L-TT, 46.8 [14.0] TT-exp, *P* = .34) scores, and overall work impairment (33.7 [26.5] TT-naïve, 22.9 [20.5] 1L-TT, 32.1 [26.7] TT-exp, *P* = .21) was observed across the 3 treatment groups (Table 2). Among patients with bowel urgency, physicians most commonly reported bowel urgency to be one of the most difficult symptoms to resolve, irrespective of treatment experience (64% TT-naïve, 54% 1L-TT, 63% TT-exp, *P* = .20), followed by bloody diarrhea (36% TT-naïve, 32% 1L-TT, 38% TT-exp, *P* = .61) and abdominal pain (26% TT-naïve, 23% 1L-TT, 27% TT-exp, *P* = .73; Table 3).

**Table 3.** Symptoms most commonly reported by physicians as difficult to resolve in patients with ulcerative colitis and bowel urgency by treatment experience.

Symptom, *n* (%)	TT-naïve (*n* = 138)	1L-TT (*n* = 143)	TT-exp (*n* = 83)	*P*-value (FE)
*n*	126	133	79	
Bowel urgency	81 (64.3)	72 (54.1)	50 (63.3)	.199
Bloody diarrhea	45 (35.7)	42 (31.6)	30 (38.0)	.606
nighttime bowel urgency	38 (30.2)	45 (33.8)	17 (21.5)	.162
Abdominal pain	33 (26.2)	30 (22.6)	21 (26.6)	.732
Non-bloody diarrhea	33 (26.2)	26 (19.5)	18 (22.8)	.444
Fatigue/tiredness	22 (17.5)	27 (20.3)	20 (25.3)	.397
Tenesmus	15 (11.9)	21 (15.8)	16 (20.3)	.269
Rectal bleeding	18 (14.3)	17 (12.8)	15 (19.0)	.460
Passing of mucus	23 (18.3)	16 (12.0)	7 (8.9)	.128
Abdominal cramps	10 (7.9)	22 (16.5)	10 (12.7)	.110
Abdominal bloating	13 (10.3)	17 (12.8)	8 (10.1)	.770
Colic	15 (11.9)	12 (9.0)	2 (2.5)	.064
Flatulence	9 (7.1)	14 (10.5)	1 (1.3)	.040

Abbreviations: FE, Fisher’s exact test; TT-naïve, patients who were currently receiving conventional therapy with a duration >3 months and had never received targeted therapy; 1L-TT, patients who were currently receiving their first targeted therapy with a duration >3 months; TT-exp, patients who were currently receiving their second or later targeted therapy with duration >3 months.

### Crohn’s Disease

Data were provided by 346 gastroenterologists for 2541 patients with CD (France; *n* = 439 [17%], Germany; *n* = 458 [18%], Italy; *n* = 414 [16%], Spain; *n* = 458 [18%] , United Kingdom; *n* = 229 [9%] and the United States; *n* = 543 [21%]). Patients with CD were stratified into 3 treatment groups and further categorized into patients with reported bowel urgency and those with no bowel urgency. Physicians reported that 17%, 13%, and 15% of TT-naïve, 1L-TT, and TT-experienced patients, respectively, were experiencing bowel urgency at the time of data collection (Table 5).

### Comparison of Patients With and Without Bowel Urgency

The demographic characteristics of the groups were similar irrespective of their bowel urgency status (Table 4). There was a significantly higher rate of moderate to severe disease among patients with versus without bowel urgency across the treatment groups (TT-naïve; 36% vs 12%, 1L-TT; 47% vs 22%, and TT-exp; 60% vs 35%, all *P* < .01; Table 4). A lower proportion of patients with bowel urgency were in remission compared to those without bowel urgency, although this only achieved statistical significance in the 1L-TT group (TT-naïve; 86% vs 93%, *P* = .15, 1L-TT; 68% vs 84%, *P* = .04, TT-exp; 60% vs 78%, *P* = .06). Patients with bowel urgency in the 1L-TT and TT-exp groups were more likely to be currently flaring than those without bowel urgency (1L-TT; 15% vs 5%, TT-exp; 29% vs 8%, both *P* < .01; Table 4). Current steroid use was significantly higher among patients with bowel urgency in the TT-naïve (45% vs 20%, *P* < .01) and 1L-TT (15% vs 5%, *P* < 0.01) groups compared to patients without bowel urgency. Mean EQ VAS scores indicated greater HRQoL impairment among patients with versus without bowel urgency for the 1L-TT group (71.6 [21.0] vs 80.5 [15.6], *P* < .01), while mean SIBDQ scores signified worse HRQoL for patients with bowel urgency irrespective of treatment experience (TT-naïve; 49.0 [8.5] vs 57.7 [10.8], 1L-TT; 47.9 [14.7] vs 54.0 [12.3], TT-exp; 45.4 [11.9] vs 53.3 [12.4], all *P* < .01). TT-naïve and TT-exp patients with bowel urgency reported greater overall work impairment compared to patients without bowel urgency (TT-naïve; 26.4 [17.5] vs 11.9 [14.9], *P* < .01, TT-exp; 27.9 [16.2] vs 17.2 [19.3], *P* = .04; Table 4).

**Table 4.** Demographic and disease characteristics of patients with Crohn’s disease by bowel urgency status and treatment experience.

	TT-naïve			1L-TT			TT-exp		
Variable	No BU (*n* = 533)	BU (*n* = 110)	*P*-value	No BU (*n* = 868)	BU (*n* = 126)	*P*-value	No BU (*n* = 344)	BU (*n* = 60)	*P*-value
*Physician-reported*									
Age, mean (SD)	39.1 (13.0)	37.8 (13.1)	.326 (*t*-test)	38.9 (13.9)	40.0 (13.0)	.371 (*t*-test)	41.4 (12.7)	44.7 (14.1)	.070 (*t*-test)
Sex, male, *n* (%)	283 (53.1)	62 (56.4)	.600 (FE)	475 (54.7)	59 (46.8)	.104 (FE)	190 (55.2)	30 (50.0)	.484 (FE)
BMI, mean (SD)	24.0 (3.5)	23.4 (2.9)	.057 (*t*-test)	24.3 (4.1)	24.0 (3.9)	.570 (*t*-test)	24.3 (4.2)	23.9 (3.5)	.590 (*t*-test)
Smoking status, *n*	495	103	.036 (CH)	835	117	.041 (CH)	327	59	.584 (CH)
Current smoker, *n* (%)	88 (17.8)	28 (27.2)		153 (18.3)	22 (18.8)		62 (19.0)	14 (23.7)	
Ex-smoker, *n* (%)	129 (26.1)	30 (29.1)		226 (27.1)	44 (37.6)		105 (32.1)	20 (33.9)	
Never smoked, *n* (%)	278 (56.2)	45 (43.7)		456 (54.6)	51 (43.6)		160 (48.9)	25 (42.4)	
*Employment status, n*	512	109	.787 (CH)	843	125	.052 (CH)	330	58	.005 (CH)
Working full-time, *n* (%)	333 (65.0)	66 (60.6)		461 (54.7)	55 (44.0)		207 (62.7)	26 (44.8)	
Working part-time, *n* (%)	58 (11.3)	11 (10.1)		102 (12.1)	25 (20.0)		37 (11.2)	4 (6.9)	
On long-term sick leave, *n* (%)	4 (0.8)	1 (0.9)		18 (2.1)	5 (4.0)		12 (3.6)	7 (12.1)	
Homemaker, *n* (%)	25 (4.9)	9 (8.3)		58 (6.9)	10 (8.0)		18 (5.5)	9 (15.5)	
Student, *n* (%)	41 (8.0)	9 (8.3)		110 (13.0)	17 (13.6)		19 (5.8)	4 (6.9)	
Retired, *n* (%)	29 (5.7)	6 (5.5)		54 (6.4)	4 (3.2)		20 (6.1)	5 (8.6)	
Unemployed, *n* (%)	22 (4.3)	7 (6.4)		40 (4.7)	9 (7.2)		17 (5.2)	3 (5.2)	
*Duration of IBD, n*	479	99	.488 (*t*-test)	795	111	.638 (*t*-test)	314	55	.245 (*t*-test)
Duration of IBD, years, mean (SD)	4.4 (5.8)	4.0 (5.4)		5.3 (6.1)	5.6 (6.5)		10.0 (7.6)	11.3 (9.0)	
*Current severity, n*	533	110	<.001 (MW)	868	126	<.001 (MW)	344	60	<.001 (MW)
Mild, *n* (%)	471 (88.4)	70 (63.6)		676 (77.9)	67 (53.2)		224 (65.1)	24 (40.0)	
Moderate/severe, *n* (%)	62 (11.6)	40 (36.4)		192 (22.1)	59 (46.8)		120 (34.9)	36 (60.0)	
*Current remission status, n*	199	49	.147 (MW)	218	38	.038 (MW)	91	30	.060 (MW)
In remission, *n* (%)	185 (93.0)	42 (85.7)		183 (83.9)	26 (68.4)		71 (78.0)	18 (60.0)	
*Current flaring status, n*	478	103	.250 (FE)	801	115	<.001 (FE)	329	58	<.001 (FE)
Flaring, *n* (%)	26 (5.4)	9 (8.7)		40 (5.0)	17 (14.8)		25 (7.6)	17 (29.3)	
*Current steroid use, n*	533	110	<.001 (FE)	868	126	<.001 (FE)	344	60	.331 (FE)
Receiving steroids, *n* (%)	105 (19.7)	50 (45.5)		44 (5.1)	19 (15.1)		28 (8.1)	7 (11.7)	
*HCP visits, n*	333	82	.575 (*t*-test)	668	106	.662 (*t*-test)	279	49	.984 (*t*-test)
HCP visits in past year, mean (SD)	2.6 (4.4)	2.9 (3.9)		3.4 (4.7)	3.6 (4.2)		4.2 (5.9)	4.2 (6.7)	
*Patient-reported*									
EQ VAS, *n*	217	55	.122 (*t*-test)	245	39	.002 (*t*-test)	97	30	.718 (*t*-test)
EQ VAS, mean (SD)	79.4 (12.8)	76.3 (13.6)		80.5 (15.6)	71.6 (21.0)		73.3 (20.8)	71.8 (13.6)	
WPAI, *n*	125	29	<.001 (*t*-test)	121	17	.373 (*t*-test)	60	17	.040 (*t*-test)
Overall work impairment, mean (SD) %	11.9 (14.9)	26.4 (17.5)		17.0 (22.3)	22.0 (15.6)		17.2 (19.3)	27.9 (16.2)	
SIBDQ, *n*	214	53	<.001 (*t*-test)	241	37	.006 (*t*-test)	96	30	.003 (*t*-test)
SIBDQ total score, mean (SD)	57.7 (10.8)	49.0 (8.5)		54.0 (12.3)	47.9 (14.7)		53.3 (12.4)	45.4 (11.9)	

Abbreviations: BMI, body mass index; BU, bowel urgency; CH, χ^2^ test; EQ VAS, EuroQol visual analog scale; FE, Fisher’s exact test; HCP, healthcare practitioner; IBD, inflammatory bowel disease; MW, Mann–Whitney test; SD, standard deviation; SIBDQ, Short Inflammatory Bowel Disease Questionnaire; TT-naïve, patients who were currently receiving conventional therapy with a duration >3 months and had never received targeted therapy; 1L-TT, patients who were currently receiving their first targeted therapy with a duration >3 months; TT-exp, patients who were currently receiving their second or later targeted therapy with duration >3 months; WPAI, work productivity and activity impairment.

### Comparison of Treatment Groups in Patients With Bowel Urgency

When comparing patients with bowel urgency across the 3 treatment groups, there was a significant difference in mean age (37.8 [13.1] TT-naïve, 40.0 [13.0] 1L-TT, 44.7 [14.1] TT-exp, *P* < .01), disease duration (4.0 [5.4] TT-naïve, 5.6 [6.5] 1L-TT, 11.3 [9.0] TT-exp, years, *P* < .01), and in employment status, with 61% of TT-naïve, 44% of 1L-TT, and 45% of TT-exp patients working full-time (*P* < .01; Table 5). No significant differences were observed between the groups for patient sex, BMI, or smoking status.

In terms of clinical characteristics, the proportion of patients with moderate to severe disease remained high among patients with bowel urgency (36% TT-naïve, 47% 1L-TT, 60% TT-exp, *P* = .01), with a high proportion of patients currently flaring (9% TT-naïve, 15% 1L-TT, 29% TT-exp, *P* < .01; Table 5). Steroid use was high among patients with bowel urgency, particularly in the TT-naïve cohort (45% TT-naïve, 15% 1L-TT, 12% TT-exp, *P* < .01). The mean number of HCP visits in the past year was similar across patients with bowel urgency (2.9 [3.9] TT-naïve, 3.6 [4.2] 1L-TT, 4.2 [6.7] TT-exp, *P* = .30). Similarly high HRQoL, as measured by mean EQ VAS (76.3 [13.6] TT-naïve, 71.6 [21.0] 1L-TT, 71.8 [13.6] TT-exp, *P* = .29) and SIBDQ (49.0 [8.5] TT-naïve, 47.9 [14.7] 1L-TT, 45.4 [11.9] TT-exp, *P* = .39) scores, and overall work impairment (26.4 [17.5] TT-naïve, 22.0 [15.6] 1L-TT, 27.9 [16.2] TT-exp, *P* = .56) was observed across the 3 treatment groups (Table 5). Among patients with bowel urgency, physicians most commonly reported this symptom to be one of the most difficult symptoms to resolve (60% TT-naïve, 58% 1L-TT, 53% TT-exp, *P* = .62), followed by non-bloody diarrhea (46% TT-naïve, 29% 1L-TT, 34% TT-exp, *P* = .03), abdominal pain (29% TT-naïve, 34% 1L-TT, 47% TT-exp, *P* = .06), and fatigue/tiredness (25% TT-naïve, 26% 1L-TT, 41% TT-exp, *P* = .06), irrespective of treatment experience (Table 6).

**Table 5.** Physician-reported patient characteristics and patient-reported outcomes in patients with Crohn’s disease and bowel urgency by treatment experience.

	CD			
Variable	TT-naive (*n* = 110)	1L-TT (*n* = 126)	TT-exp (*n* = 60)	*P*-value
*Physician-reported*				
Age, mean (SD)	37.8 (13.1)	40.0 (13.0)	44.7 (14.1)	.006 (*t*-test)
Sex, male, *n* (%)	62 (56.4)	59 (46.8)	30 (50.0)	.338 (FE)
BMI, mean (SD)	23.4 (2.9)	24.0 (3.9)	23.9 (3.5)	.291 (*t*-test)
Smoking status, *n*	103	117	59	.577 (CH)
Current smoker, *n* (%)	28 (27.2)	22 (18.8)	14 (23.7)	
Ex-smoker, *n* (%)	30 (29.1)	44 (37.6)	20 (33.9)	
Never smoked, *n* (%)	45 (43.7)	51 (43.6)	25 (42.4)	
Employment status, *n*	109	125	58	.005 (CH)
Working full-time, *n* (%)	66 (60.6)	55 (44.0)	26 (44.8)	
Working part-time, *n* (%)	11 (10.1)	25 (20.0)	4 (6.9)	
On long-term sick leave, *n* (%)	1 (0.9)	5 (4.0)	7 (12.1)	
Homemaker, *n* (%)	9 (8.3)	10 (8.0)	9 (15.5)	
Student, *n* (%)	9 (8.3)	17 (13.6)	4 (6.9)	
Retired, *n* (%)	6 (5.5)	4 (3.2)	5 (8.6)	
Unemployed, *n* (%)	7 (6.4)	9 (7.2)	3 (5.2)	
Duration of IBD, *n*	99	111	55	<.001 (*t*-test)
Duration of IBD, years, mean (SD)	4.0 (5.4)	5.6 (6.5)	11.3 (9.0)	
Current severity, *n*	110	126	60	.012 (MW)
Mild, *n* (%)	70 (63.6)	67 (53.2)	24 (40.0)	
Moderate/severe, *n* (%)	40 (36.4)	59 (46.8)	36 (60.0)	
Current remission status, *n*	49	38	30	.029 (MW)
In remission, *n* (%)	42 (85.7)	26 (68.4)	18 (60.0)	
Current flaring status, *n*	103	115	58	.002 (FE)
Flaring, *n* (%)	9 (8.7)	17 (14.8)	17 (29.3)	
Current steroid use, *n*	110	126	60	<.001 (FE)
Receiving steroids, *n* (%)	50 (45.5)	19 (15.1)	7 (11.7)	
HCP visits, *n*	82	106	49	.303 (*t*-test)
HCP visits in past year, mean (SD)	2.9 (3.9)	3.6 (4.2)	4.2 (6.7)	
*Patient-reported*				
EQ VAS, *n*	55	39	30	.289 (*t*-test)
EQ VAS, mean (SD)	76.3 (13.6)	71.6 (21.0)	71.8 (13.6)	
WPAI, *n*	29	17	17	.558 (*t*-test)
Overall work impairment, mean (SD) %	26.4 (17.5)	22.0 (15.6)	27.9 (16.2)	
SIBDQ, *n*	53	37	30	.387 (*t*-test)
SIBDQ total score, mean (SD)	49.0 (8.5)	47.9 (14.7)	45.4 (11.9)	

Abbreviations: BMI, body mass index; BU, bowel urgency; CH, χ^2^ test; EQ VAS, EuroQol visual analog scale; FE, Fisher’s exact test; HCP, healthcare practitioner; IBD, inflammatory bowel disease; MW, Mann–Whitney test; SD, standard deviation; SIBDQ, Short Inflammatory Bowel Disease Questionnaire; TT-naïve, patients who were currently receiving conventional therapy with a duration >3 months and had never received targeted therapy; 1L-TT, patients who were currently receiving their first targeted therapy with a duration >3 months; TT-exp, patients who were currently receiving their second or later targeted therapy with duration >3 months; WPAI, work productivity and activity impairment.

**Table 6.** Symptoms most commonly reported by physicians as difficult to resolve in patients with Crohn’s disease and bowel urgency by treatment experience.

Symptom, *n* (%)	TT-naive (*n* = 110)	1L-TT (*n* = 126)	TT-exp (*n* = 60)	*P*-value (FE)
*n*	106	120	59	
Bowel urgency	64 (60.4)	69 (57.5)	31 (52.5)	.621
Non-bloody diarrhea	49 (46.2)	35 (29.2)	20 (33.9)	.026
Abdominal pain	31 (29.3)	41 (34.2)	28 (47.5)	.061
Fatigue/tiredness	26 (24.5)	31 (25.8)	24 (40.7)	.063
Night-time bowel urgency	28 (26.4)	30 (25.0)	12 (20.3)	.678
Abdominal bloating	17 (16.0)	20 (16.7)	10 (16.9)	.986
Abdominal cramps	20 (18.9)	18 (15.0)	7 (11.9)	.474
Colic	22 (20.8)	17 (14.2)	3 (5.1)	.024
Bloody diarrhea	16 (15.1)	15 (12.5)	10 (16.9)	.703
Flatulence	13 (12.3)	14 (11.7)	3 (5.1)	.307
Postprandial bowel movements	11 (10.4)	11 (9.2)	3 (5.1)	.505
Tenesmus	3 (2.8)	17 (14.2)	3 (5.1)	.005
Arthralgia	2 (1.9)	7 (5.8)	10 (16.9)	<.001

Abbreviations: FE, Fisher’s exact test; TT-naïve, patients who were currently receiving conventional therapy with a duration >3 months and had never received targeted therapy; 1L-TT, patients who were currently receiving their first targeted therapy with a duration >3 months; TT-exp, patients who were currently receiving their second or later targeted therapy with duration >3 months.

## Discussion

This study, conducted in 5 major European countries and the United States, aimed to assess the impact of bowel urgency in patients with moderate to severe UC and patients with CD (any severity). We found that, despite being treated for their UC or CD, a substantial proportion of patients continued to experience bowel urgency, irrespective of receiving conventional or TT.

For many years, UC and CD have been treated mainly with 5-ASAs, corticosteroids, and immunosuppressants. The recent development of biologics with novel mechanisms of action and small-molecule drugs has improved the treatment and thus the prognosis of IBD patients.^38^ However, the efficacy of currently available treatments in resolving inflammation is limited in that some patients may have an inadequate response, lose response over time, or may not tolerate a given drug, thus resulting in discontinuation of therapy or suboptimal treatment. As such, a significant unmet need remains as suboptimal treatment is associated with higher rates of surgery, hospitalization, and/or prolonged corticosteroid use as well as impaired HRQoL.^39^

Our study found that, despite receiving treatment for their UC or CD for more than 3 months, 17%-26% of UC and 13%-17% of CD patients, depending on their experience with TT, experienced persistent bowel urgency, with physicians most commonly reporting bowel urgency as one of the most difficult to treat symptoms among this subset of patients. Overall, patients with bowel urgency were less likely to be in remission and more likely to have moderately or severely active UC or CD, were more likely to be flaring, and more likely to be receiving steroids than those without bowel urgency. Patients with bowel urgency also reported worse HRQoL and greater levels of overall work impairment than those without. These findings were largely consistent, irrespective of the patient’s treatment group.

A substantial clinical and HRQoL burden remained, irrespective of treatment experience, when comparing patients with bowel urgency. However, an increased burden of disease was demonstrated among the TT-exp group, where a higher proportion of UC and CD patients were currently flaring, and among CD patients, there was a lower proportion of patients in remission and a higher proportion of moderate to severe patients. While the increased clinical burden among the TT-exp group is largely unsurprising, since these patients are likely to have received multiple treatment regimens due to their lack of response, this finding highlights the unmet need among these difficult-to-treat patients.

Despite some differences across the treatment groups, the rate of remission, as measured by the total Mayo score and CDAI, among patients experiencing bowel urgency was relatively high overall (20%-30% UC, 60%-85% CD). In the development of validated patient-reported outcomes tools for UC and CD patients, bowel urgency was found to be a relevant symptom to measure response to treatment in both UC and CD.^40^ In another recent study, bowel urgency was found to be 1 of the 4 key symptoms, on top of the conventional patient-reported outcomes, that may be helpful in predicting endoscopic mucosal healing status in UC.^41^ Since the present study has demonstrated that patients with bowel urgency experience a substantial clinical and HRQoL burden, this suggests the need for further consideration around how remission in UC and CD is defined and highlights the relevance of tools which consider major burdensome symptoms such as bowel urgency, as a more comprehensive measure of disease activity.

While this study identified that physicians perceive bowel urgency as being one of the most difficult symptoms to resolve among this subset of patients, due to the fact that completion of the patient self-reported questionnaire was voluntary we did not investigate the patient perspective of this symptom as data were not available for all patients. Bowel urgency is commonly underreported by physicians, due to both lack of awareness and the absence of validated instruments to quantify severity and effect, therefore this study may underreport the burden of bowel urgency. A survey of patients with IBD that used choice-based conjoint analysis to estimate the relative importance of 4 common symptoms found that bowel urgency was the most important symptom to patients, followed by abdominal pain and blood in stools. Bowel urgency associated with incontinence received particularly high scores and was perceived to be more than 3 times as important as bowel urgency without incontinence.^11^ Hence, not only is bowel urgency viewed as a symptom that is highly difficult to resolve by physicians, but it is also regarded by patients as one of the key symptoms to be addressed.

Recent evidence- and consensus-based recommendations for selecting the goals for treat-to-target strategies in patients with IBD have now identified the most relevant long-term achievable treatment targets to be clinical remission, endoscopic healing, restoration of HRQoL, and absence of disability. Symptomatic relief has been determined as an immediate goal since this is rated highest by patients in studies.^42^ Hence, there exists a significant unmet need for the development of new therapeutic options to address key burdensome symptoms, such as bowel urgency, among patients with IBD.

### Limitations

This was a non-interventional study, with physicians completing forms on consecutive consulting patients with IBD to mitigate selection bias. Eligible patients were screened and selected by physicians, and it is therefore recognized that patients who were visiting physicians more often are more likely to have been included in the study.

It should be noted that the survey was designed to facilitate understanding of real-world clinical practice, and thus physicians could only report on data they had to hand at the time of the consultation. Therefore, this represents the evidence they had when making any clinical treatment and other management decisions at that consultation. These patients were encouraged, but not mandated, to complete all forms such that base sizes fluctuate across different variables. It is also acknowledged that the study relies on the accuracy of physicians when completing each record form and the willingness of patients to complete their questionnaires. To minimize the risk of collecting inaccurate data, the questionnaires were relatively short and user-friendly with electronic routing and logic applied to ensure no contradictions in responses. In addition, forms and questionnaires were completed at the time of consultation to reduce recall bias.

Finally, the cross-sectional design of this study prevents any conclusions about causal relationships, although identification of significant associations is possible. This study, nevertheless, involved a high number of physicians, working in different settings, across different geographical regions, thereby ensuring that the sample is likely to be representative of the overall population of physicians and their consulting patients with IBD.

## Conclusion

This real-world study found that a substantial proportion of patients with moderate to severe UC and patients with CD experience bowel urgency, irrespective of receiving either conventional or TT. Moreover, patients with bowel urgency experience an increased clinical and HRQoL burden compared to patients without bowel urgency, with the burden remaining substantial across patients with differing levels of TT experience. Despite current treatment options, new therapeutic strategies are needed to address the most challenging symptoms in people living with IBD.

## Data Availability

All data. ie, methodology, materials, data, and data analysis, that support the findings of this survey are the intellectual property of Adelphi Real World. All requests for access should be addressed directly to H.K. at hannah.knight@adelphigroup.com. H.K. is an employee of Adelphi Real World.
